# Antifungal and Ichthyotoxic Sesquiterpenoids from *Santalum album* Heartwood

**DOI:** 10.3390/molecules22071139

**Published:** 2017-07-08

**Authors:** Tae Hoon Kim, Tsutomu Hatano, Keinosuke Okamoto, Takashi Yoshida, Hiroshi Kanzaki, Michiko Arita, Hideyuki Ito

**Affiliations:** 1 Department of Food Science and Biotechnology, Daegu University, Gyeongsan 38453, Korea; skyey7@daegu.ac.kr; 2 Faculty of Pharmaceutical Sciences, Okayama University, Tsushima, Okayama 700-8530, Japan; hatano-t@cc.okayama-u.ac.jp (T.H.); k-oka@xd6.so-net.ne.jp (K.O.); xp769b@bma.biglobe.ne.jp (T.Y.); 3 Faculty of Agriculture, Okayama University, Tsushima, Okayama 700-8530, Japan; hkanzaki@okayama-u.ac.jp; 4 Faculty of Health and Welfare Science, Okayama Prefectural University, Soja, Okayama 719-1197, Japan; chamichan@gj9.so-net.ne.jp

**Keywords:** *Santalum album* L., Santalaceae, α-santalol, ichthyotoxicity, antifungal effect, *Trichophyton rubrum*, inulavosin, antimitotic activity

## Abstract

In our continuing study on a survey of biologically active natural products from heartwood of *Santalum album* (Southwest Indian origin), we newly found potent fish toxic activity of an *n*-hexane soluble extract upon primary screening using killifish (medaka) and characterized α-santalol and β-santalol as the active components. The toxicity (median tolerance limit (TLm) after 24 h at 1.9 ppm) of α-santalol was comparable with that of a positive control, inulavosin (TLm after 24 h at 1.3 ppm). These fish toxic compounds including inulavosin were also found to show a significant antifungal effect against a dermatophytic fungus, *Trichophyton rubrum*. Based on a similarity of the morphological change of the immobilized *Trichophyton* hyphae in scanning electron micrographs between treatments with α-santalol and griseofulvin (used as the positive control), inhibitory effect of α-santalol on mitosis (the antifungal mechanism proposed for griseofulvin) was assessed using sea urchin embryos. As a result, α-santalol was revealed to be a potent antimitotic agent induced by interference with microtubule assembly. These data suggested that α-santalol or sandalwood oil would be promising to further practically investigate as therapeutic agent for cancers as well as fungal skin infections.

## 1. Introduction

*Santalum* species (sandalwood) (Santalaceae) are evergreen parasitic trees and include about 25 species that distribute in India, Indonesia, Malaysia and Australia [1]. Their essential oil, sandalwood oil, has been old used as a noble perfume upon producing incense sticks, deodorants, cosmetics and aromatherapy agents as well as medicines [2]. Previous phytochemical studies on sandalwood oil have revealed the occurrence of numerous phenylpropanoids [3] and sesquiterpenoids [4,5,6,7,8,9] including α-santalol [10,11] and β-santalol [10,12]. Among various biological properties reported for the oil and α-santalol are antiviral [13], anticarcinogenic [14], neuroplastic [10,15,16] and antitumor effects [17,18]. In our previous study on exploring bioactive natural products, we had investigated heartwood chips of *Santalum album* L. of South West Indian (Mysore) origin, which is regarded as the best sandalwood in quality, and demonstrated the characterization of new neolignans [19] and sesquiterpenoids, and their evaluation for in vitro and in vivo antitumor-promoting effects [20,21]. Further investigation of the *S. album* chips in the present study revealed the occurrence and characterization of ichthyotoxic components in the hexane-soluble extract upon screening with ichthyotoxic assay [22] to killifish (*Oryzias latipes*; Japanese name, medaka). Naturally occurring toxic substances to small fish have been proved to be generally nontoxic to warm-blooded animals, including human beings, upon oral administration, and also to often possess a variety of other biological properties beneficial to human health, such as antitumor-, antifungal-, antiulcer- and antitumor-promoting effects [22,23,24,25,26,27]. Thus, this assay has been considered to be useful as a simple and convenient preliminary screening test to find diverse bioactive natural products. Based on this background for the ichthyotoxic substances, we assessed antifungal effect of the piscicidal components and related constituents isolated from the sandalwood extract against dermatophytic fungus, *Trichophyton rubrum.* Here we describe these results and a possible antifungal mechanism.

## 2. Results and Discussion

**Figure 1:**
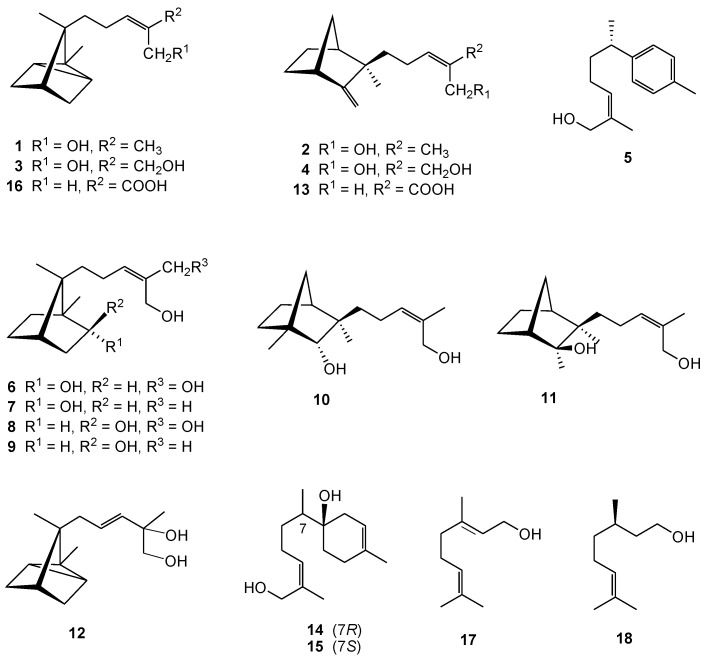
Structures of the tested compounds isolated from *S. album* of Indian Origin.

Test compounds, used for assessment of ichthyotoxic, antifungal and antimitotic effects using previously reported methods [22,28,29], were obtained from *S. album* heartwood. The methanol extract of heartwood partitioned with *n*-hexane and EtOAc, to afford the respective extracts. In the ichthyotoxic assay, fish toxic activity was only exhibited by *n*-hexane extract. Compounds isolated from the *n*-hexane extract are summarized as follows: α-santalol (**1**), β-santalol (**2**), α-santaldiol (**3**) [30], β-santaldiol (**4**) [30], (+)-α-nuciferol (**5**) [31,32], (2*R*,7*R*)-2,12,13-trihydroxy-10-campherene (**6**) [21], (2*R*,7*R*)-2,12-dihydroxy-10(*Z*)-campherene (**7**) [21], (2*S*,7*R*)-2,12,13-trihydroxy-10-campherene (**8**) [21], (2*S*,7*R*)-2,12-dihydroxy-10(*Z*)-campherene (**9**) [21], (2*R*,3*R*)-10(*Z*)-sandalnol (**10**) [21], (2*S*,3*R*)-10(*Z*)-neosandalnol (**11**) [21], 9(*E*)-11-hydroxy-α-santalol (**12**) [20], 10(*E*)-β-santalic acid (**13**) [20], (1*R*,7*R*)-1,12-dihyroxybisabola-3,10-diene (**14**) [20], (1*R*,7*S*)-1,12-dihyroxybisabola-3,10-diene (**15**) [20], α-santalenoic acid (**16**) [21], geraniol (**17**) [20], and (+)-citronellol (**18**) [20] (Figure 1).

Among these isolates, major components, α-santalol (**1**), β-santalol (**2**), α-santaldiol (**3**), β-santaldiol (**4**), and (+)-α-nuciferol (**5**), were tested for their ichthyotoxic activity. α-Santalol (**1**) and β-santalol (**2**) showed potent ichthyotoxicity to killifish with median tolerance limit (TLm) (after 24 h) values, 1.9 ppm and 5.0 ppm, respectively, while α-santaldiol (**3**), β-santaldiol (**4**), and (+)-α-nuciferol (**5**) were nontoxic at 10 ppm. The potency of **1** was comparable to those of fish toxins, inulavosin (TLm 1.3 μg/mL) [26] and buddledin B (TLm 1.2 μg/mL) [27] used as positive controls. Although other minor active components may occur in the extract, the ichthyotoxicity of the sandalwood oil was mainly responsible for **1** and **2**.

Inulavosin was originally isolated as ichthyotoxic substance from *Inula nelvosa* and found to have antibacterial activity [26] and melanogenesis inhibitory effect [32]. In this study, we additionally found the antifungal property of inulavosin and the *n*-hexane extract of sandalwood against *Trichophyton rubrum* which causes superficial mycoses commonly known as tinea infections. In order to characterize the active components of the extract, antifungal effect of santalols and other isolates along with inulavosin against *T. rubrum* was evaluated by the disc diffusion method [28] comparing with griseofulvin as a positive control. As shown in Table 1, α-santalol (**1**) demonstrated substantial activity with the minimum inhibitory concentration (MIC) value of 12.5 μg/disc, which was comparable with that of inulavosin (10 μg/disc). Compared with **1**, the other compounds showed weaker activity against the tested human pathogen with MIC values ranging from 25.0 to 125 μg/disc.

**Figure 2:**
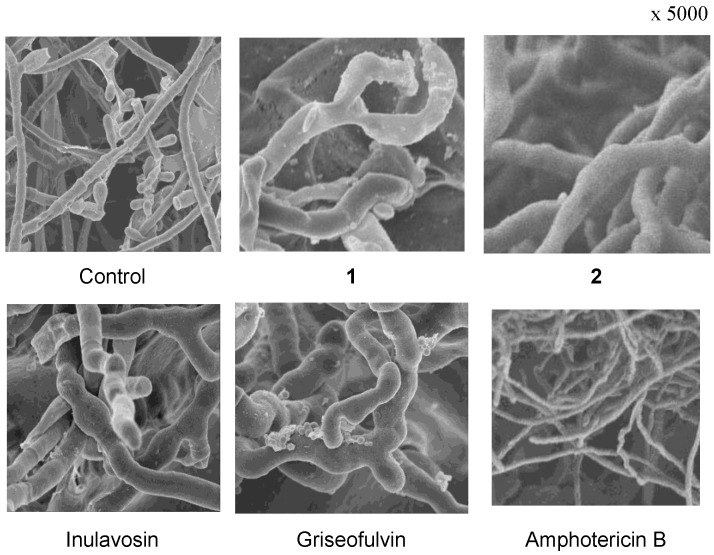
Scanning electron micrographs of compound-induced morphological change against *T. rubrum*.

The scanning electron microscopy (SEM) features of the immobilized hyphae of *T. rubrum* treated with inulavosin indicated a morphological change of the terminal hyphae with curing and swelling which is very similar to that of griseofulvin, suggesting their similarity in the action mechanism (Figure 2). Griseofulvin is fungistatic antibiotic and one of its mechanisms is proposed as an interference with the synthesis of certain components of the fungal cell walls, such as chitin [33]. The effect on cell wall synthesis is represented by morphological alteration, leading to abnormal development of the terminal hyphae [34] that become enlarged, thickened and curled. SEMs of α-santalol (**1**) and β-santalol (**2**), the potent antifungal agents in sandalwood, also showed a change of the terminal hyphae with curing and swelling, similar to those of griseofulvin and inulavosin (Figure 2). These results suggested that **1** and **2**, as well as inulavosin, are fungistatic, possibly induced by a mechanism similar to that of griseofulvin.

**Figure 3:**
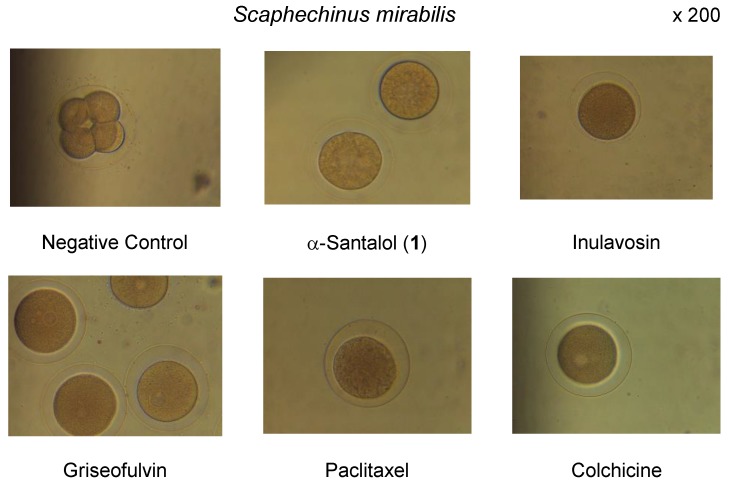
Antimitotic activity of α-santalol (**1**) and some natural products in sea urchin embryos.

Underlying antifungal property of griseofulvin has been proposed to inhibit mitosis by breaking the structure of the mitotic envelope, thus stopping cell division at the metaphase stage [35]. Tubulin is the major protein component of microtubules that are involved in a wide number of cellular function such as motility, division, shape maintenance, and intracellular transport. Interference with microtubule assembly, either by inhibition of tubulin polymerization or by blocking microtubule disassembly, leads to an increase in the number of cell metaphase arrest. Inhibition of microtubule function using targeting agents is a validated approach to anticancer therapy [36,37]. The tubulin interactive effect of α-santalol (**1**), inulavosin and griseofulvin was estimated by observation of cell division in sexually mature fertilized eggs of three kinds of sea urchins, *Hemicentrotus pulcherrimus*, *Anthocidaris crassispina* and *Scaphechinus mirabilis*. As shown in Table 2 and Figure 3, these concerned compounds showed potent tubulin (de)polymerization-inhibiting activity at metaphase, with efficacy comparable to those of the positive controls, paclitaxel [35] and colchicine. Thus, the tubulin (de)polymerization inhibitory effect of griseofulvin was consistent with the reported hypothetic mechanism [37]. Antifungal effect of the santalols against *T. rubrum* was also suggested to be induced by their antimitotic ability.

In the present study, we found the occurrence of fish toxic substances by the ichthyotoxic assay using medaka and characterized α-santalol and β-santalol which are major sesquiterpenoids in *n*-hexane soluble portion, as active components. The fish toxic substances, santalols and inulavosin (positive control), were also revealed to have antifungal activity against the dermatophytic fungus, *T. rubrum*. Treatment with α-santalol and inulavosin showed morphological changes of the hyphae in SEMS, which were similar to that upon treatment with griseofulvin. A possible mechanism of their antifungal activity was suggested to be due to interference of fungous cell wall synthesis or their antimitotic effect, which was substantiated by the inhibition of the cell division in sea urchin embryos.

## 3. Materials and Methods

### 3.1. Isolation of the Test Compounds

The heartwood of *S. album* (1.53 kg) was extracted with MeOH at room temperature. The combined crude MeOH extracts (73.1 g) were suspended in 20% MeOH (2 L), then partitioned in turn with *n*-hexane (3 × 2 L) and EtOAc (3 × 2 L) to afford dried *n*-hexane-soluble (16.4 g), EtOAc-soluble (27.1 g), and H_2_O-soluble (17.5 g) residues. The *n*-hexane extract exhibited significant ichthyotoxicity against test fishes when evaluated at 40 μg/mL, while the other extracts were all nontoxic at the same concentration. Upon performing chromatographic separation, the fractions were monitored with normal-phase and reversed-phase HPLC. The tested compounds listed in Table 1 were obtained from our previous investigation [20,21] and their structures are illustrated in Figure 1. The purity of the tested compounds was confirmed by TLC and HPLC analyses.

### 3.2. Ichthyotoxic Assay

The assay was conducted as previously reported using medaka (*Oryzias latipes*). A test solution was prepared by adding an acetone solution (0.5 mL) of the compounds of known concentration into aerated water (100 mL). Five groups containing different known concentration for each compound were estimated in order to determine the 50% lethal dose (TLm) of fishes after 24 h via straight-line graphical interpolation. A control experiment (0.5 mL acetone only) was conducted under the same conditions.

### 3.3. Assay for Antifungal Activity

The dermatophytes employed in this study were obtained from Fujita Gakuin. *Trychophyton rubrum* FH01 was maintained through monthly subculturing on Sabouraud Dextrose Agar (SDA) at room temperature. The antifungal activity against *T*. *rubrum* was measured using the paper-disk agar diffusion method. Dried paper disks (diameter: 8 mm) containing test material were placed on SDA plates seeded with fungi and incubated at 30 °C for 5 days. The clear inhibition zone outside the paper disk was measured in millimeters. Griseofulvin was used as the antifungal standard.

### 3.4. Scanning Electron Microscopy (SEM)

Immobilized hyphae of *T*. *rubrum* FH01 were subjected to SEM observation. The hyphas obtained by cultivating SDA containing 0.1% peptone broth and the immobilized hyphae using polyvinyl alcohol (PVA) gel beads were fixed with 2% glutaraldehyde for 60 min, washed with 0.1 M phosphate buffer (pH 7.2) five times, and dehydrated with an ethanol gradient from 20% to 99%, followed by 100% *t*-butanol. The specimens were freeze-dried and coated with platinum using a Hitachi ES-2030 and Hitachi E-1030, respectively. SEM observation was performed using a Hitachi SEM S-4500.

### 3.5. Measurement of Tubulin Interactive Effects

Sexually mature sea urchins were collected during the breeding season (*Hemicentrotus pulcherrimus*; January–March, *Anthocidaris crassispina*; June, *Scaphechinus mirabilis*; October–November) from the intertidal marsh near Ushimado Marine Laboratory in Okayama Prefecture. The eggs and sperm were obtained by 0.5 M KCl shedding. The sperm shed from the genital papilla was collected with a glass capillary (dry sperm). The eggs were placed in filtered sea water and gently agitated. After some minutes, the eggs near the surface and the bottom were removed by decantation and suction. This process promised a better fertilization rate and synchronous development of the eggs. The eggs were inseminated at room temperature with the sperm suspension. Development of the control eggs was checked under a microscope until the swimming blastula stage (24 h after fertilization). In the first cell cycle of the normal eggs, metaphase was observed at 65–70 min after fertilization, and the first cleavage occurred at 85–90 min after fertilization. Samples shown in Table 2 with each known concentration were dissolved in a small amount of 20 μL MeOH:DMSO (1:1) and diluted with 1 mL sea water. The fertilized eggs were treated with sample solutions at 35 min after fertilization and left for 40 min. The eggs were subjected to microscopic analysis, and the treated eggs were compared with the control eggs at metaphase using SEM.

## 4. Conclusions

α-Santalol (**1**), a major component in most species of the *Santalum* genus, has been known to have a variety of physiological activities, including neuroleptic property, antitumor and chemopreventive effects of cancers under in vitro and in vivo bioassay systems [39,40]. This study thus provided an additional evidence for the usefulness of santalols that possess antifungal and antimitotic properties, like griseofulvin, as well as small fish toxicity. The sandalwood oil or α-santalol would thus be promising to further investigate practically as new therapeutic agents for fungal infections and of cancer chemoprevention. In addition, the present study provided further evidence of the effectiveness of the ichthyotoxic assay to find diverse bioactive natural products beneficial to human health from natural sources, although relationships between the ichthyotoxicity and the other activities are not clear.

## Figures and Tables

**Table 1:** Antifungal activity of constituents isolated from *S. album* against *Trichophyton rubrum*.

Compounds	MIC (μg/disc) ^a^	Compound	MIC (μg/disc)
**1**	12.5	**11**	31.3
**2**	25.0	**12**	62.5
**3**	50.0	**13**	125.0
**4**	25.0	**14**	125.0
**5**	31.3	**15**	125.0
**6**	250.0	**16**	62.5
**7**	62.5	**17**	62.5
**8**	250.0	**18**	62.5
**9**	62.5	Inulavosin	10.0
**10**	62.5	Griseofulvin	0.5

**^a^** MIC (minimum inhibitory concentration) was defined as the concentration of 0.5 mm inhibitory zone produced by the tested compound. Values represent average obtained from a minimum of three experiments.

**Table 2:** Inhibitory effects of the selected compounds against mitosis of sea urchin embryos.

	MIC (μg/mL) ^a^		
Compounds	*Hemicentrotus* *pulcherrimus*	*Anthocidaris* *crassispina*	*Scaphechinus* *mirabilis*
**1**	25	12.5	12.5
**2**	>50	>50	>50
**3**	>50	>50	>50
**4**	>50	>50	>50
**5**	>50	>50	>50
Inulavosin	50	25	50
Griseofulvin	3.13	3.13	3.13
Paclitaxel ^b^	10 ^c^	nt	25
Colchicine ^b^	nt ^d^	nt	50

^a^ The first cleavage of sea urchin embryos was blocked when treated with a concentration higher than MIC. ^b^ Positive control substances. ^c^ Reported inhibition values [38]. ^d^ nt: not tested.
